# Reverse Vaccinology and Immunoinformatic Assisted Designing of a Multi-Epitopes Based Vaccine Against Nosocomial *Burkholderia cepacia*

**DOI:** 10.3389/fmicb.2022.929400

**Published:** 2022-06-28

**Authors:** Noorah Alsowayeh, Aqel Albutti, Samia T. Al-Shouli

**Affiliations:** ^1^Department of Biology, College of Education (Majmaah), Majmaah University, Al-Majmaah, Saudi Arabia; ^2^Department of Medical Biotechnology, College of Applied Medical Sciences, Qassim University, Buraydah, Saudi Arabia; ^3^Immunology Unit, Pathology Department, College of Medicine, King Saud University, Riyadh, Saudi Arabia

**Keywords:** pseudomonas cepacia, reverse vaccinology, immunoinformatic, molecular docking, MD simulations

## Abstract

*Burkholderia cepacia* is a Gram-negative nosocomial pathogen and is considered as a troublesome bacterium due to its resistance to many common antibiotics. There is no licensed vaccine available to prevent the pathogen infections, thus making the condition more alarming and warrant the search for novel therapeutic and prophylactic approaches. In order to identify protective antigens from pathogen proteome, substantial efforts are put forth to prioritized potential vaccine targets and antigens that can be easily evaluated experimentally. In this vaccine design investigation, it was found that *B. cepacia* completely sequenced proteomes available in NCBI genome database has a total of 28,966 core proteins. Out of total, 25,282 proteins were found redundant while 3,684 were non-redundant. Subcellular localization revealed that 18 proteins were extracellular, 31 were part of the outer membrane, 75 proteins were localized in the periplasm, and 23 were virulent proteins. Five proteins namely flagellar hook protein (FlgE), fimbria biogenesis outer membrane usher protein, Type IV pilus secretin (PilQ), cytochrome c4, flagellar hook basal body complex protein (FliE) were tested for positive for antigenic, non-toxic, and soluble epitopes during predication of B-cell derived T-cell epitopes. A vaccine peptide of 14 epitopes (joined together *via* GPGPG linkers) and cholera toxin B subunit (CTBS) adjuvant (joined to epitopes peptide *via* EAAAK linker) was constructed. Binding interaction of the modeled vaccine with MHC-I, MHC-II, and Toll-like receptor 4 (TLR-4) immune receptors was studied using molecular docking studies and further analyzed in molecular dynamics simulations that affirms strong intermolecular binding and stable dynamics. The maximum root mean square deviation (RMSD) score of complexes in the simulation time touches to 2 Å. Additionally, complexes binding free energies were determined that concluded robust interaction energies dominated by van der Waals. The total energy of each complex is < −190 kcal/mol. In summary, the designed vaccine showed promising protective immunity against *B. cepacia* and needs to be examined in experiments.

## Introduction

The term “antibiotic resistance” refers to bacteria ability to withstand the action of antibiotics to which they had previously been susceptible (Tacconelli et al., 2018; Albekairi et al., 2022b). Bacteria have developed antibiotic resistance rapidly in the past 25 years, and the situation is very alarming now (Hasan and Al-Harmoosh, 2020). In addition, new resistant strains are surfacing faster and spreading more rapidly than ever before (Chokshi et al., 2019). During this process, new mutations progress in bacterial genomes and resistance genes are acquired through environmental exposure. The overuse and misuse of antibiotics is the main pressure that force bacterial to evolve and adapt its self to ever changing environmental conditions (Kulik et al., 2019; Albekairi et al., 2022a). The antibiotic resistance genes are disseminated primarily through extra chromosomal plasmids from one organism to another. The antimicrobial resistance is increasing to a frightening level, while on the other hand, new antibiotic development is very slow (MacLean and San Millan, 2019). Therefore, new therapeutic and prophylactic approaches are urgently required to address antibiotic resistance crisis.

Antibiotic resistant bacterial pathogen can be managed by developing safe vaccines (Jansen and Anderson, 2018; ud-din et al., 2022). Compared to antibiotics, vaccine resistance and associated allergic reactions are less. A vaccine is a biological preparation that induces protective adaptive immune responses against the pathogen antigen(s). Vaccines have a long success history in managing infectious organisms. The first smallpox vaccine was developed by Edward Jenner using the cowpox virus to provide protection against variola virus (Smith, 2011). There are many types of approaches that can be used to develop good vaccines. Louis Pasteur used the weekend form of the bacillus as a vaccine to treat anthrax (Opal, 2010). Sabin and Stalk developed a vaccine for polio using the Pasteur vaccine principles (Naz et al., 2015). The Bacillus Calmette Guerin (BCG) vaccine was developed against *Mycobacterium tuberculosis* (Bali et al., 2015). Despite of conventional vaccinology success, its application for unculturable pathogen and non-conserved antigens is limited. Fewer culture-based vaccines have been developed as a result. Traditional vaccine development is time-consuming and expensive (Rappuoli, 2001).

The Burkholderia cepacia complex (BCC) consists Gram-negative bacteria that produce catalase and do not ferment lactose (LiPuma, 2005). *B. cepacia* is a human pathogen that causes pneumonia most often in immunocompromised people with underlying lung diseases (such as cystic fibrosis) (Datta et al., 2020). The pathogen is described originally as a plant pathogen but become a major cause of infections especially hospitals. This pathogen is still poorly understood in terms of its virulence and ability to cause pathogenesis in humans (Goldmann and Klinger, 1986). It is intrinsically resistant to number of antibiotics such as aminoglycosides and colistin (Mahenthiralingam et al., 2005). Most patients who contract *B. cepacia* are merely colonized, but has the capacity to cause serious infections such as those caused by surgical and burn wounds, bacteremia, meningitis, pneumonia, peritonitis, and urinary tract infections. The organism can survive in a variety of solutions, disinfectants, medications, and even antiseptics (Rojas-Rojas et al., 2018). Moreover, no licensed vaccine is available in the market to prevent *B. cepacian* infections.

Recent advances in vaccine technology have led to computational reverse vaccinology, where antigenic surface proteins are determined from a genome dataset (Rappuoli, 2000; Suleman et al., 2021; Alharbi et al., 2022b). Vaccine for meningococcal serogroup B (4CMenB) was developed using reverse vaccinology (Serruto et al., 2012). Considering the importance of reverse vaccinology in identifying potential vaccine targets in pathogens genomic data, the method was used herein to highlight protein targets and antigenic epitopes that can be prioritized against the pathogen to assist experimentalists in vaccine development. Moreover, a multi-epitopes vaccine construct is designed to overcome limitations of simple peptide vaccine. Further, biophysics approaches are utilized to show binding and dynamics of the vaccine with immune receptors.

## Methodology

**FIGURE 1:**
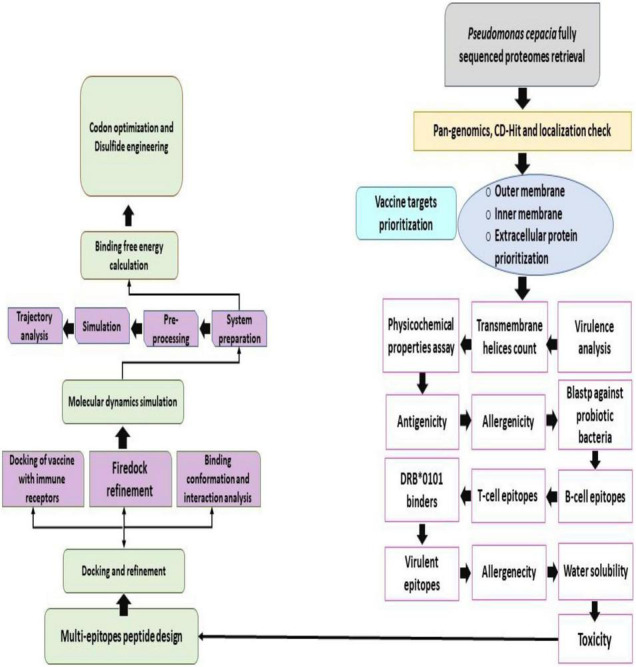
Work flow of the steps done in the study to design a multi-epitopes vaccine against *B. cepacian*.

The different steps used for vaccine construct design are highlighted in Figure 1.

### Pan-Genomics and Subcellular Localization

The NCBI database was used to collect proteomic data of *B. cepacian* (Coordinators, 2017). Pan-proteome analysis was performed on the proteomes to determine pathogen core sequences. In order to perform the pan-proteome analysis, we used the Bacterial Pan Genome Analysis tool (BPGA) (Chaudhari et al., 2016). A CD-Hit clustering process was used to remove redundant proteins from the core sequence obtained through BPGA (Fu et al., 2012). We set the threshold at 0.5 and as such 50% similar sequences were discarded. PSORTb 3.0 subcellular localization prediction tool was used to predict the subcellular localization of selected non-redundant proteins (Yu et al., 2010). Only extracellular, outer membrane and periplasmic proteins were opted for further downward analysis. These proteins are exposed to the host immune systems, and have antigenic sequences capable of stimulating strong immune responses (Ahmad et al., 2019).

### Selection of Vaccine Candidates

Afterward, several filters were applied on the selected outer membrane, extracellular and periplasmic proteins to perform selection of potential vaccine targets. Proteins that play a significant role in pathogenesis are prime targets for vaccine design (Ahmad et al., 2019; Alharbi et al., 2022a). A BLASTp search was conducted against the core virulent factor database (VFDB) (Liu et al., 2019) using output proteins from the subcellular localization step, and those with sequence identities of ≥ 30% and bit scores ≥ 100 were selected (Qamar et al., 2021). Afterward, the ProtParam tool was used to determine the physico-chemical properties of selected virulent proteins (ProtParam, 2017). It has been suggested that proteins having a molecular weight of ≤ 110 kDa serve as ideal candidates for vaccine development as they can be easily purified (Ismail et al., 2020). HMMTOP 2.0 (Tusnady and Simon, 2001) was used to predict the transmembrane helices of the proteins. The selection was made if a protein has 1or 0 transmembrane helices (Rizwan et al., 2017). As a next step, shortlisted proteins were BLASTp against a reference human proteome with the “taxonomic ID: 9606” and those with a sequence identity ≤ 30% were selected (Naz et al., 2015). All homologous proteins were discarded in order to avoid autoimmune reactions. A threshold of 0.4 was used by VaxiJen 2.0 to determine the antigenicity of proteins (Ong et al., 2021). The antigenic proteins are good vaccine targets because they are antibodies generator. After selecting antigenic proteins, it was necessary to determine whether they had adhesive properties because proteins that possess adhesive properties can trigger pathogenic pathways. A SPAAN server was used to test the adhesive properties of proteins (Sachdeva et al., 2005). AllerTOP2.0 was applied further to evaluate allergic proteins among the adhesive proteins (Dimitrov et al., 2013). The homology of non-allergic proteins to *Lactobacillus rhamnosus, L. casei*, and *L. johnsonii* was examined by BLASTp (Ahmad et al., 2019).

### B-Cell and T-Cell Epitopes Prediction

Bepipred Linear Epitope Prediction 2.0 of Immune Epitope Database (IEDB) was used to predict linear B-cell epitopes at threshold of 0.5 (Vita et al., 2015; Jespersen et al., 2017). The resultant B-cell epitopes were utilized to predict common peptides showing strong binding to class I and class II alleles of the major histocompatibility complex (MHC) (Dhanda et al., 2019). This was one using IEDB T-cell epitopes prediction tools. Those with minimum percentile score were regarded as strong binders and subjected to MHCPred 2.0 analysis (Guan et al., 2003). In this anlaysis, only those epitopes with an IC50 value of ≤ 100 nM were selected as they show strong binding to the highly prevalent DRB*0101 allele. VaxiJen (Doytchinova and Flower, 2007), AllerTOP 2.0 (Dimitrov et al., 2013), and VirulentPred (Garg and Gupta, 2008) were again used to validate predicted epitopes antigenicity, allergenicity, and virulence, respectively. Innovagen, which is a peptide solubility calculator, was used to determine the solubility of peptides. Peptides that show good solubility in water were selected for further investigation. Using ToxinPred (Gupta et al., 2013), non-toxic epitopes were selected (Barh et al., 2013).

### Chimeric Vaccine Design

The shortlisted epitopes were then used chimeric vaccine design. As single peptide vaccine has weak immunogenicity, this problem was overcome by fusing all epitopes with GPGPG linkers to stimulate strong immune responses that can effectively combat the pathogen (Malonis et al., 2019). A cholera toxin B (CTB) adjuvant was additionally added to epitopes peptide using EAAAK linker. SCRATCH protein predictor (Cheng et al., 2005) was used to predict the tertiary structure of the chimeric vaccine. The refinement of the structure was performed by Galaxy web refine tool (Heo et al., 2013).

### Blind Docking Analysis

Blind docking between designed chimeric vaccine and immune cell receptors was PatchDock server (Schneidman-Duhovny et al., 2005), The TLR-4, MHC-I, or MHC-II pdb structures were retrieved from protein data bank (PDB) using 4 digit code of 4G8A, 1I1Y, and 1KG0, respectively. FireDock server was used for refinement of docked complexes generated through PatchDock (Mashiach et al., 2008). In each case, top complex that showed lowest global energy was considered and evaluated further. The binding between vaccine and receptors was examined using UCSF Chimera 1.13.1 (Pettersen et al., 2004).

### Molecular Dynamics Simulation Assay

Molecular dynamics simulation was used to analyze the stability and dynamics of docked complexes (Karplus, 2002). AMBER20 was used for this purpose (Case et al., 2020). The systems were first subjected to energy minimization, followed by heating, equilibrium and production (Ahmad et al., 2019). The production run was conducted for 100 ns. The systems preparation was done using antechamber program (Wang et al., 2001). FF14SB force field was employed to parameterize the complexes (both receptors and vaccine molecules) (Case et al., 2014). In energy minimization, steepest descent and conjugate gradient algorithms were used. In temperature, the systems temperature was gradually increased to 310 K. During production, SHAKE algorithm was used to apply constrain on hydrogen bonds while Langevin algorithm was used to maintain temperature (Izaguirre et al., 2001; Kräutler et al., 2001). CPPTRAJ module of the AMBER was used to measure systems intermolecular docked stability (Roe and Cheatham, 2013). The XMGRACE was considered for plotting root mean square deviation (RMSD) and root mean square fluctuation (RMSF) graphs (Maiorov and Crippen, 1994; Turner, 2005; Qamar et al., 2021).

### Estimation of Binding Free Energy

In order to calculate the solvation and gas free energies associated with the interactions of the vaccine with all three receptors, the MMPBSA.py module was employed (Miller et al., 2012). A total of 100 frames were picked from molecular dynamics simulation trajectories and used in calculation of binding free energy.

### Codon Adaptation and Cloning

In order to achieve high expression of the vaccine, the vaccine sequence was converted into DNA sequences and then codon usage was adapted to the *E. coli* K-12 strain to attain high vaccine expression JCAT (Java Codon Adaptation Tool) server was used for this process (Grote et al., 2005). GC-content and codon adaptation index (CAI) was used to determine the expression level of the vaccine.

### Disulfide Engineering

Introducing disulfide bonds to the vaccine structure was done to make the vaccine structure stabile. This also protect the vaccine enzyme degradable prone regions (Dombkowski et al., 2014). Disulfide engineering was carried out by Design 2.0 (Craig and Dombkowski, 2013). Only high energy residue pairs (> 0 kcal/mol) were mutated to cysteine.

## Results

### Retrieval of *Burkholderia cepacia* Proteomes

A total of 7 complete sequenced proteomes of *B. cepacia* were obtained from NCBI genome database. Only fully sequenced proteomes were considered to achieve consistency in the results. Information regarding each strain genome size and GC contents were also accessed as tabulated in Table 1.

**TABLE 1:** Completely sequenced *B. cepacia* strains, genome size and GC% contents.

S. No	Strain	Size (Mb)	GC%
1	*B. cepacia* CMCC (B)23005	8.64	66.68
2	*B. cepacia* BC16	8.37	66.76
3	*B. cepacia* DDS 7H-2	8.15	67.06
4	*B. cepacia* MINF_4A-sc-2280433	7.94	67.11
5	*B. cepacia* ATCC 25416	8.57	66.57
6	*B. cepacia* GG4	6.47	66.71
7	*B. cepacia* JBK9	8.48	66.81

### Bacterial Pan-Genomics

**FIGURE 2:**
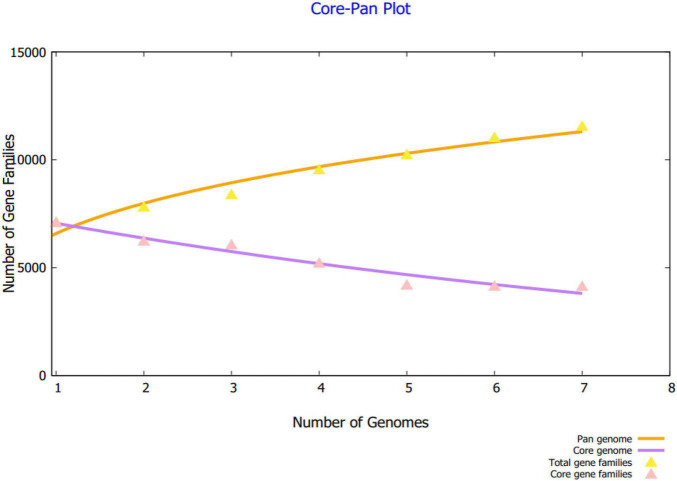
The plot of the core pan of seven *B. cepacia* strains. The number of gene families in respective pathogen strain is also provided.

Using BPGA, the core sequences shared by all the strains were extracted from the complete proteomes. Along with that, BPGA enabled the separation of strain-specific proteins as well as dispensable proteins. As core proteins are present in all of the sequenced strains, they are involved in core function of the pathogen and thus vital for pathogen survival, growth and functionality (Chaudhari et al., 2016). Considering this significance role of core proteins in the pathogen biology, they are termed as attractive vaccine targets. The core pan plot illustrating the number of gene families in each strain of the pathogen is given in Figure 2.

### CD-HIT Analysis

**FIGURE 3:**
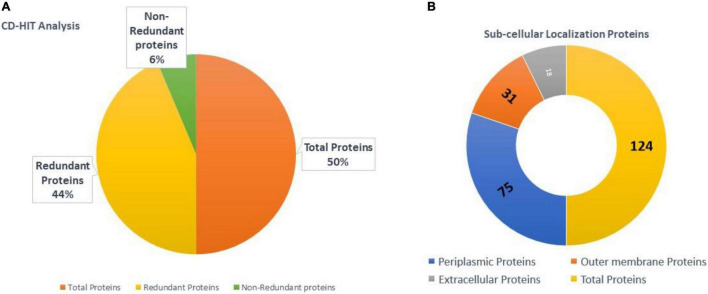
**(A)** Total, non-redundant proteins, and redundant proteins of *B. cepacia*. **(B)** Categorization of surface proteins obtained.

A CD-HIT analysis was used to separate redundant and non-redundant proteins in the core proteome. There were 28,966 total proteins in proteomic data. Approximately, 3,684 proteins were found non-redundant and 25,282 to be redundant as shown in Figure 3A. The redundant proteins are the products of gene duplication event and are present in more than one copy in the genomes/proteome. Thus, considering them in afterward analysis is less scientifically sound. On the other hand, non-redundant proteins are promising vaccine targets due to their prime role in pathogen essential pathways and functions (Sanober et al., 2017).

### Subcellular Localization Analysis

A subcellular localization strategy was adopted in order to predict proteins part of the pathogen exoproteome and secretome. The surface exposed proteins play a vital role in pathogen ability to invade, adhere to, and multiply within host cells. Additionally, these proteins are disclosed to contain multiple antigenic regions capable of eliciting strong immune responses (Barh et al., 2013). Most proteins were located in cytoplasm, 18 were extracellular, 31 were outer membrane, 75 were in periplasm, and some were unknown. In total, 124 were surface-localized proteins as illustrated in Figure 3B.

### Selection of Good Vaccine Targets

As part of the analysis, the virulence factor database (VFDB) was used to identify virulent proteins of *B. cepacia*. Virulent proteins trigger stronger immune responses and critical disease pathway initiation, making them good targets for vaccine development (Naz et al., 2015). The study found total of 19 virulent proteins as presented in Table 2. Further, estimation of transmembrane helices in the proteins revealed all 19 proteins to have either 0 or 1 transmembrane helices, which to its easy ability to be easily purified in experimental analysis. All of the proteins also unveiled to have less molecule weight than the threshold and thus are considered to be good targets because of their easy cloning and expression (Ahmad and Azam, 2018). The proteins are also non-allergic and adhesive in nature.

**TABLE 2:** List of good vaccine targets filtered after several vaccine checks.

Extracellular proteins	Bit score	Sequence identity	HMMTOP	TMHMM	Molecular weight (kDa)	Antigenicity	Allergenicity	Adhesive (> 0.5)
core/1086/1/Org1_Gene5543	337	39%	1	0	58.34	Yes	Non-allergen	Adhesive
core/2596/1/Org1_Gene5174	625	75%	0	0	42.78			
core/8300/1/Org1_Gene1809	113	45%	1	0	17.25			
**Outer membrane proteins**						Yes	Non-allergen	Adhesive
core/274/1/Org1_Gene7050	464	35%	0	0	94.19			
core/407/1/Org1_Gene2987	202	35%	1	0	81.17			
core/1146/1/Org1_Gene2828	259	35%	0	0	57.50			
core/1616/1/Org1_Gene1810	251	34%	0	0	51.59			
**Periplasmic proteins**								
core/973/1/Org1_Gene3623	465	46%	0	0	62.55	Yes	Non-allergen	Adhesive
core/1378/1/Org1_Gene6277	367	42%	0	0	56.86			
core/2630/1/Org1_Gene2121	120	44%	0	0	41.29			
core/3033/1/Org1_Gene1368	207	45%	0	1	38.31			
core/3736/1/Org1_Gene2308	132	30%	0	0	33.65			
core/4580/1/Org1_Gene1573	104	30%	0	0	27.14			
core/6422/1/Org1_Gene1807	182	40%	1	1	22.58			
core/7180/1/Org1_Gene2836	139	41%	0	0	11.53			
core/7662/1/Org1_Gene3427	211	65%	1	1	33.89			
core/9884/1/Org1_Gene1032	122	92%	0	0	58.34			
core/37/4/Org4_Gene4766	742	35%	0	0	42.78			
core/4251/4/Org4_Gene4249	285	83%	0	0	17.25			

By using ProtParm tool, physico-chemical properties were evaluated for selected vaccine proteins. This analysis is helpful to investigate the vaccine targets experimentally. Only, 16 proteins were selected in this phase as they fulfill all the filters of physico-chemical stage. Different parameters such as aliphatic index, GRAVY, composition of amino acids, and instability index were estimated as given in Table 3. All proteins scored negatively on the GRAVY index, indicating their hydrophilic nature. The proteins were also found thermally stable and have accepted PI value.

**TABLE 3:** Physico-chemical properties of good vaccine targets.

Extracellular proteins	Amino acid	PI	Instability index	Aliphatic index	GRAVY
core/1086/1/Org1_Gene5543	557	4.77	25.73	62.12	−0.297
core/2596/1/Org1_Gene5174	414	6.28	18.93	69.61	−0.342
core/8300/1/Org1_Gene1809	170	6.18	8.36	77.06	0.056
**Outer membrane proteins**					
core/274/1/Org1_Gene7050	881	8.92	26.1	74.88	−0.294
core/407/1/Org1_Gene2987	781	7.78	38.18	93.05	−0.134
core/1146/1/Org1_Gene2828	547	8.48	33.85	88.63	−0.137
core/1616/1/Org1_Gene1810	486	7.79	39.07	94.34	−0.01
**Periplasmic proteins**					
core/973/1/Org1_Gene3623	583	6.29	31.57	82.18	−0.148
core/1378/1/Org1_Gene6277	511	6.58	28.93	70.12	−0.472
core/3033/1/Org1_Gene1368	389	9.97	33.21	76.68	−0.228
core/3736/1/Org1_Gene2308	350	8.95	43.94	85.63	−0.303
core/4580/1/Org1_Gene1573	315	5.78	29.84	91.14	0.02
core/6422/1/Org1_Gene1807	248	8.37	45.92	89.31	−0.071
core/7180/1/Org1_Gene2836	217	8.98	20.18	74.84	−0.18
core/9884/1/Org1_Gene1032	114	4.94	31.01	80.61	0.175
core/4251/4/Org4_Gene4249	329	9.47	25.11	70.58	−0.189

Probiotics bacteria aid to inhibit the growth and survival of microbes that are harmful to the body. Vitamins such as biotin and vitamin K2 are produced by these organisms. In order to avoid accidental inhibition of probiotic bacteria, filtered proteins were used in BLASTp search was performed against multiple probiotic bacteria. By doing so, only 5 proteins were selected for further study and remaining proteins were discarded. The proteins also revealed non-homology to the human host and they are likely not to generate any autoimmune responses (Table 4).

**TABLE 4:** Homology check of proteins against probiotic bacteria and human proteome.

Extracellular proteins	*Lactobacillus casei* (taxid:1582)	*Lactobacillus jhonsoni (taxid:33959)*	*Lactobacillus rhamnosus (taxid:47715)*	Human (taxid:9606)
Core/2596/1/Org1_Gene5174 Flagellar hook protein FlgE				
**Outer membrane proteins**				
Core/274/1/Org1_Gene7050 Fimbrial biogenesis outer membrane usher protein				
Core/1146/1/Org1_Gene2828 Type IV pilus secretin PilQ	No similarity	No similarity	No similarity	No similarity
**Periplasmic proteins**				
Core/7180/1/Org1_Gene2836 Cytochrome c4				
Core/9884/1/Org1_Gene1032 Flagellar hook basal body complex protein FliE				

### Epitopes Prediction

**FIGURE 4:**
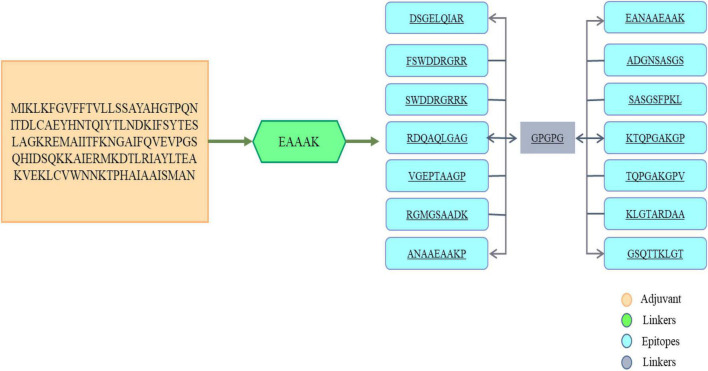
The primary amino acids sequence of vaccine. To link the antigenic B-cell derived T-cell epitopes, GPGPG linkers were used as shown in gray block. At the amino terminal of the multi-epitope’s peptide, an adjuvant (cholera toxin B subunit) was attached shown in orange box while the EAAAK linker is shown in green color box.

In order to select B-cell epitopes of score greater than the cut-off, the proteins were first subjected to B-cell epitopes prediction. The predicted epitopes are shown in Table 5. Further analysis of B-cell epitopes was done to predict MHC-I and MHC-II epitopes. For prediction of MHC epitopes, reference alleles were used in T-cell prediction. T-cell epitopes with low percentile rank were considered good binders to MHC alleles. The MHC-Pred server was used further to determine which epitopes showed IC50 values for the DRB*0101 gene. This DRB belongs to the human leukocyte antigen II family and is widely distributed throughout the world. Hence, epitopes showing good binding to DRB*0101 can produce strong and specific immune responses (Naz et al., 2019). All predicted epitopes were also non-allergenic, antigenic, water soluble and non-toxic. The screened 14 soluble and non-toxic epitopes were used in the design of a multi-epitopes as shown in Figure 4.

**TABLE 5:** T cell epitopes predicted from B cells.

B-cell epitopes	MHC-I epitopes	Percentile score	MHC-II epitopes	Percentile score	MHCPred IC50 Value (nM)
TDSGELQIARTEFAQVE	TDSGELQIAR	3.4	TDSGELQIARTEFAQV	3.6	54.7
VGRWHFRHNGSFSWDDRGRRKY	FSWDDRGRRK	3.6	GSFSWDDRGRRKY	0.82	4.18
					69.82
MQARDQAQLGAGAASVSRQR	RDQAQLGAGA	14	QARDQAQLGAGAAS	1.9	76.38
KDSVGEPTAAGPA	SVGEPTAAGP	7.1	SVGEPTAAGPA	6.5	41.78
RCDARGMGSADADKTGPEAAGAGSTRQP	RGMGSAADK	13	ARGMGSADADKTG	18	53.21
VAEANAAEAAKPDLDR	EANAAEAAKP	8.1	VAEANAAEAAKPD	4.4	38.02
					79.62
ADGNSASGSFPKLAGQHPEY	ADGNSASGSF	3.9	ADGNSASGSFPKLA	8	84.14
	SASGSFPKL	1.8	SASGSFPKLAGQHPEY	18	62.66
KTQPGAKGPVRTNAVMVGFASALSAD	KTQPGAKGPV	5.5	KTQPGAKGPVRTNA	13	36.06
					99.54
GSQTTKLGTARDAATVPIGQKIYRGGIA EKGVPA	KLGTARDAA	10	KLGTARDAATVPIG	6.9	25.23
	GSQTTKLGT	12	GSQTTKLGTARDAATV	18	52.72

### Chimeric Vaccine Construct

Peptide vaccines are poor immunogenic and therefore the predicted epitopes were joined to design a multi-epitope vaccine construct (Reche et al., 2014). The immunodominant epitopes were linked using GPGPG linkers and the resultant peptide is joined to an adjuvant cholera toxin B subunit *via* EAAAK linker. The said linkers are rigid in nature and keep the epitopes and adjuvant molecule separated and stop epitopes folder over each other (Ismail et al., 2020). This physical separation of epitopes allows the host immune simulation system to easily recognize and process the epitopes. The cholera toxin B subunit is safe and has stronger affinity for monosialotetrahexosylganglioside present on variety of antigen presenting cells, B-cells, and macrophages and thus facilitates the antigenic peptide access to the immune system (Baldauf et al., 2015). The multi-epitopes vaccine is thus producing more rigorous immune responses that are specific and accurate.

### 3D Vaccine Structure and Refinement

**FIGURE 5:**
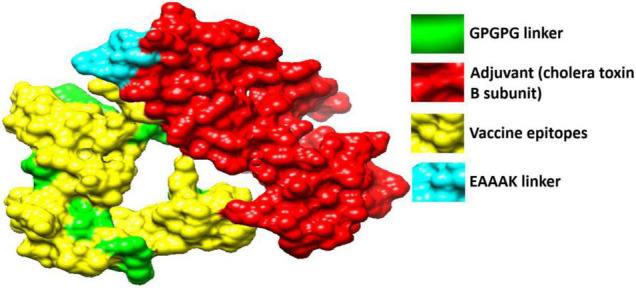
3D structure of the designed vaccine. The red color represents the adjuvant (cholera toxin B subunit), yellow color is for vaccine epitopes, cyan blue color denotes EAAAK while the green color is for GPGPG linkers.

Figure 5 shows the tertiary structure of the chimeric vaccine that was modeled by *ab initio* method as no appropriate template was present to be the vaccine structure on homology principle. The vaccine was then subjected to loop modeling where loop regions were modeled into secondary structure elements. The loop model structure was then refined for structure errors and the most refined model was selected (Heo et al., 2013). Model 1 was opted due to its good galaxy energy of -4200.50, absence of poor rotamers, low clash score, and highest percentage of residues in Rama favored.

### Molecular Docking

**FIGURE 6:**
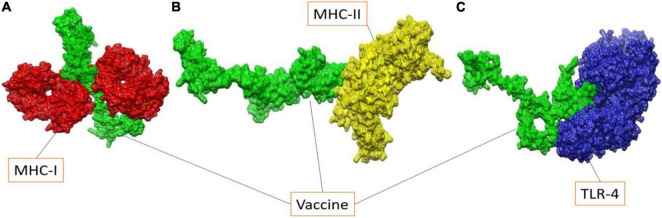
Illustration of docked vaccine to MHC-I molecule **(A)**, MHC-II molecule **(B)** and TLR-4 molecule **(C)**.

In case of MHC-I, solution 10 was regarded as the best representative intermolecular binding conformation as it has the lowest global binding energy (-12.94 kJ mol^–1^) which can be split into attractive van der Waals (-34.63 kJ mol^–1^), atomic contact energy (4.12 kJ mol^–1^), and hydrogen bond energy (-2.10 kJ mol^–1^). Solution 8 from MHC-II and solution 5 from TLR-4 has total global binding of -5.21 kJ mol^–1^ and -7 kJ mol^–1^, respectively. The FireDock refinement results for MHC-I and MHC-II, as well as TLR-4 are summarized in Supplementary Tables 1–3, respectively. The docked vaccine conformations with the different immune receptors used are given in Figure 6.

Proper immune response depends on the interaction between vaccines and host immune cells. Using the protein-peptide docking approach, these interactions between vaccine constructs and MHC-I were determined, and specific residue-wise interactions of MHC-I, MHC-II and TLR-4 were found in the UCSF chimera as shown in Table 6.

**TABLE 6:** Vaccine constructs interactions with different immune receptors.

	MHC-I		MHC-II			TLR-4		
Glu212	Asn24	Val93	Asn124	Met36	Phe26	Gln39	His159	Val316
Arg202	Trp107	Lys48	Asp159	Cys15	Gly125	Trp26	Cys95	Arg69
Leu270	Asp98	Asp129	Ser95	Val75	Asp43	Ser55	Glu154	Ile301
Phe109	Arg108	Tyr67	Glu166	Ile106	Leu215	Phe122	Asp 38	Arg106
Pro235	Asp106	Val152	Trp153	Gly20	Cys212	Ala137	Leu180	His229
Ile7	Tyr257	Gln262	Pro178	Ile148	Gln130	Arg87	Phe217	Pro34

### Molecular Dynamic Simulation

**FIGURE 7:**
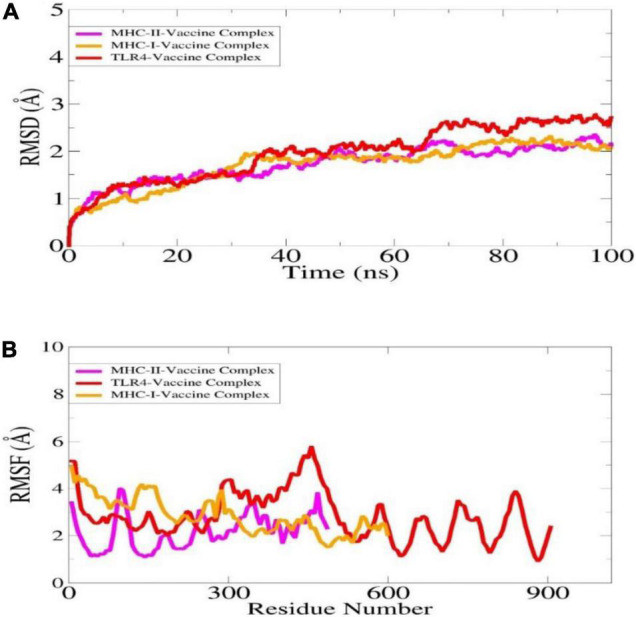
Statistical analysis of MD simulation trajectories is illustrated here: RMSD **(A)**, RMSF **(B)**.

The dynamics of complexes were investigated in order to validate intermolecular conformational stability and interactions. The structure stability was determined through root mean square deviation (RMSD), which is used to investigate global structure changes in the superimposed snapshots (Figure 7A). It was found that the complexes are quite stable along the simulation time and no major structure deviations were noticed. The RMSD of the systems are within 3 angstrom range.

Additionally, the complexes stability was evaluated through root mean square fluctuation (RMSF) (Figure 7B). Both ends of the complexes were found flexible while the complexes overall remained stable. The high flexibility of the ends is due to flexible loops which confer structure instability to the systems however, it does not affect overall vaccine binding to the receptors. The affirm that the vaccine binding to the immune receptors is strong, and the epitopes are exposed which further ensured that the epitopes might be easily recognized and processed by the host immune system cells. This further interprets the vaccine epitopes may generate stronger immune reactions.

### Estimation of Binding Free Energies

Binding free energies calculation of the docked complexes were calculated using MM/PBSA and MM/GBSA approaches. In MM-GBSA the total binding free energies of TLR-4-vaccine complex, MHC-I-vaccine complex, and MHC-II-vaccine complex for MM-GBSA were -301 kcal/mol, -250 kcal/mol, and -197 kcal/mol, respectively as given in Table 7. Similarly in MM-PBSA, it has been found that TLR-4-vaccine complex has net binding free energy of -294.64 kcal/mol while MHC-I-vaccine has -245.86 kcal/mol and MHC-II-vaccine complex net binding energy of -192.99 kcal/mol. It was further revealed that both van der Waals and electrostatic energies are favorable in complexes formation.

**TABLE 7:** Binding free energies of complexes analyzed with MM-GBSA/MM-PBSA methods.

Energy parameter	TLR-4-vaccine complex	MHC-I-vaccine complex	MHC-II-vaccine complex
**MM-GBSA**			
VDWAALS	−212.00	−184.00	−171.00
EEL	−135.00	−98.00	−59.00
Delta G gas	−347	−282	−230
Delta G solv	46.00	32.00	33.00
Delta Total	−301	−250	−197
**MM-PBSA**			
VDWAALS	−212.00	−184.00	−171.00
EEL	−135.00	−98.00	−59.00
Delta G gas	−347	−282	−230
Delta G solv	52.36	36.14	37.01
Delta total	−294.64	−245.86	−192.99

### Codon Optimization

During this process, the sequence of the model vaccine construct was first reverse translated into DNA sequence by using Java Codon Adaptation Tool (JCat) to calculate the codon adaptation index (CAI) and GC percentage rates of the cloned vaccine construct as the maximum level of expression in the *E. coli* expression system. The GC contents of the designed vaccine is 58.2% and CAI value is 1, which is considered ideal for high expression.

### Disulfide Engineering

**FIGURE 8:**
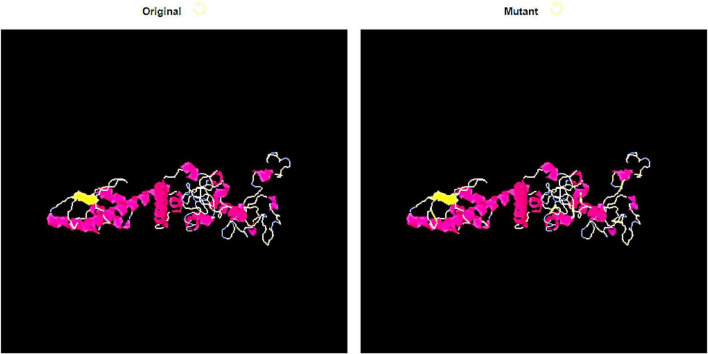
Disulfide engineering of the designed vaccine molecule. Both wild (left) and mutated structure (right) is shown. The yellow sticks in mutated structure are residues replaced with cysteine.

Disulfide engineering is a useful approach to mutate vaccine residues that are unstable from energy perspective and are pronce to host enzymatic degredation. It also enhance vaccine folded conformation stability by achieving low entropy (Dombkowski et al., 2014). Cysteine residues were added replacing unstable residues. The residues that were mutated have high unfavorable binding energy. The mutated residues can be seen in Figure 8 as yellow sticks. The following 9 residues pair: Ser16-Ala19, Ala17-Asn36, Ala31-His34, Lys44-Val71, Lys55-Gln70, Met58-Ala67, Gly211-Ala214, Ala217-Pro224, and Lys293-Asp229 were mutated to cystein in disulfide engineering.

## Discussion

Antibiotic resistance is a natural mechanism by which bacteria make itself adaptable to the changing environmental conditions. The bacterial resistance to antibiotics has reached to a very dangerous level and strains resistance to several and all commonly used antibiotics are now routinely reported across the globe (Hutchings et al., 2019). This not only length human mortality rate but also increase disease burden and pressured health care systems (Tacconelli et al., 2017; Hutchings et al., 2019). *B. cepacia*, which is a Gram-negative nosocomial human pathogen is responsible for several diseases in particular pneumonia most often in people who have immunodeficiencies or lung diseases (like cystic fibrosis) (Mahenthiralingam et al., 2005). The pathogen has become one of the most common causes of infections in hospitals. The bacteria resist many antibiotics such as cephalosporins, aminoglycosides, fluroquinolones, and trimoxazole. So far, no efficacious vaccine is present to prevent *B. cepacian* infections (Devanga Ragupathi and Veeraraghavan, 2019). None of the vaccine candidate evaluated experimentally has shown sterilizing immunity.

The introduction of fast sequencing technologies and as a result genomic data is added to databases enabled researchers to discover new vaccine candidates that were harder to detect in traditional vaccine development (Moriel et al., 2008; Naz et al., 2021). The identification of putative surface-associated antigens can be done using advanced bioinformatic approaches (Yero et al., 2021). It has been shown that reverse vaccinology is a safe, effective, and reliable method for identifying surface-associated proteins from the pathogen genome without the need to cultivate the microbes (Yero et al., 2021). An effective meningococcal vaccine has been developed using the reverse vaccinology method (Abdullah et al., 2021). A number of studies have indicated that pan-genomic reverse vaccinology (PGRV) is more effective compared to conventional reverse vaccinology because it screens highly conserved targets (Fatima et al., 2021; Ullah et al., 2021; Gul et al., 2022). Therefore, pan-genomics studies were integrated with reverse vaccinology and immunoinformatic to map potential B and T-cell epitopes in *B. cepacian* genome.

In contrast to simple peptide based vaccine, a multi-epitopes vaccine against antibiotic resistant *B. cepacia* is designed herein. Good vaccine targets were prioritized using several properties such as antigenicity, immunogenicity, non-homology to human host and probiotic bacteria, non-allergenicity, and localization on the pathogen surface. The properties listed above are based on literature and highly desirable for designing a chimeric vaccine (Qamar et al., 2020; Ismail et al., 2021). Further, immunoinformatic approaches were used for predicting B and T-cell epitopes and only safe and powerful antigens were shortlisted to be used in a chimeric vaccine design. The vaccine construct designed in this study comprises five proteins flagellar hook protein (FlgE), fimbria biogenesis outer membrane usher protein, Type IV pilus secretin (PilQ), cytochrome c4, flagellar hook basal body complex protein (FliE). A total of 14 epitopes have been predicted from selected proteins that share common sequences with ability to stimulate stronger immune responses. In addition to allergenicity and solubility analysis, we also examined toxicity and allergenicity of the epitopes. Cholera toxin B subunit adjuvant was further combined with predicted B-cell derived from T-cells epitopes peptide.

The binding ability of the designed multi-epitopes vaccine with different immune receptors was further examined using biophysics approaches. The findings reported that the designed vaccine showed robust interactions with the receptors and are engaged by several intermolecular interactions. The stable intermolecular dynamics validated the efficient representation of epitopes to the immune cells for processing of immune signaling pathways.

## Conclusion

The study investigates the development of a multi-epitopes vaccine against *B. cepacia*. Several computer aided vaccine approaches are being explored, such as reverse vaccination, subtractive proteomics, immunoinformatic, and biophysical analysis. We predicted several vaccine targets; flagellar hook protein (FlgE), fimbria biogenesis outer membrane usher protein, type IV pilus secretin (PilQ), cytochrome c4, flagellar hook basal body complex protein (FliE). Total of 14 soluble and non-toxic epitopes were predicted from the above proteins and were used in the design of a multi-epitopes vaccine. The designed vaccine showed robust interactions with the host immune receptors and found to show stable conformation in simulation time. Thus, it can be concluded that the predicted epitopes and designed vaccine construct might provide protection against the pathogen.

## Data Availability Statement

The original contributions presented in this study are included in the article/Supplementary Material, further inquiries can be directed to the corresponding author/s.

## Author Contributions

NA and AA contributed to conception and design of the study. SA-S collected the data. NA performed the analysis. AA wrote the first draft of the manuscript. NA and SA-S wrote sections of the manuscript. All authors contributed to manuscript revision, read, and approved the submitted version.

## Conflict of Interest

The authors declare that the research was conducted in the absence of any commercial or financial relationships that could be construed as a potential conflict of interest.

## Publisher’s Note

All claims expressed in this article are solely those of the authors and do not necessarily represent those of their affiliated organizations, or those of the publisher, the editors and the reviewers. Any product that may be evaluated in this article, or claim that may be made by its manufacturer, is not guaranteed or endorsed by the publisher.
