# Supplementary material for: Reverse Vaccinology and Immunoinformatic Assisted Designing of a Multi-Epitopes Based Vaccine Against Nosocomial Burkholderia cepacia
Source: Front Microbiol. 2022 Jun 28;13:929400. doi: 10.3389/fmicb.2022.929400 (PMC9297367; doi:10.3389/fmicb.2022.929400)
Supplement: Supplementary file 1 [file Data_Sheet_1.docx]

**Supplementary File**

# Reverse Vaccinology and Immunoinformatic Assisted Designing of a Multi-epitopes based Vaccine against Nosocomial *Pseudomonas cepacia*

Noorah Aslowayeh^1,†,*^, Aqel Albutti^2,†,*^, Samia T. Al-Shouli^3^

# Department of Biology, College of Education (Majmaah), Majmaah University, Al-Majmaah,11952, Saudi Arabia; [n.alsowayeh@mu.edu.sa](mailto:n.alsowayeh@mu.edu.sa)

Department of Medical Biotechnology, College of Applied Medical Sciences, Qassim University, Buraydah, Saudi Arabia; [as.albutti@qu.edu.sa](mailto:as.albutti@qu.edu.sa)

# Pathology department, Immunology Unit, College of Medicine, King Saud University, PO BOX 2925, Riyadh 11461, Saudi Arabia; [salshouli@ksu.edu.sa](mailto:salshouli@ksu.edu.sa)

**†** These authors contributed equally to this study.

# *Correspondence: [n.alsowayeh@mu.edu.sa](mailto:n.alsowayeh@mu.edu.sa) (N.A.); [as.albutti@qu.edu.sa](mailto:as.albutti@qu.edu.sa) (A.A.)

**S-Table 1.** FireDock server generated top 10 docked models of vaccines to MHC-I.

| **Rank** | [**Solution Number**](http://bioinfo3d.cs.tau.ac.il/FireDock/bin/showRes.pl?id=1i1yMHCI.pdb_Refinedmodel4.pdb_52_53_20_19_3_122&from=1&to=20&sortBy=1) | [**Global Energy**](http://bioinfo3d.cs.tau.ac.il/FireDock/bin/showRes.pl?id=1i1yMHCI.pdb_Refinedmodel4.pdb_52_53_20_19_3_122&from=1&to=20&sortBy=2) | [**Attractive VdW**](http://bioinfo3d.cs.tau.ac.il/FireDock/bin/showRes.pl?id=1i1yMHCI.pdb_Refinedmodel4.pdb_52_53_20_19_3_122&from=1&to=20&sortBy=3) | [**Repulsive VdW**](http://bioinfo3d.cs.tau.ac.il/FireDock/bin/showRes.pl?id=1i1yMHCI.pdb_Refinedmodel4.pdb_52_53_20_19_3_122&from=1&to=20&sortBy=4) | [**ACE**](http://bioinfo3d.cs.tau.ac.il/FireDock/bin/showRes.pl?id=1i1yMHCI.pdb_Refinedmodel4.pdb_52_53_20_19_3_122&from=1&to=20&sortBy=5) | [**HB**](http://bioinfo3d.cs.tau.ac.il/FireDock/bin/showRes.pl?id=1i1yMHCI.pdb_Refinedmodel4.pdb_52_53_20_19_3_122&from=1&to=20&sortBy=6) |
| --- | --- | --- | --- | --- | --- | --- |
| 1 | 10 | -12.94 | -34.63 | 14.44 | 4.12 | -2.10 |
| 2 | 3 | -3.50 | -24.23 | 24.04 | 6.18 | -1.65 |
| 3 | 4 | 4.99 | -0.00 | 0.00 | 0.00 | 0.00 |
| 4 | 5 | 13.72 | -2.82 | 0.00 | 3.89 | 0.00 |
| 5 | 8 | 18.17 | -5.75 | 0.86 | 1.71 | 0.00 |
| 6 | 6 | 27.45 | -11.44 | 5.92 | 13.01 | -1.22 |
| 7 | 9 | 28.29 | -13.71 | 15.09 | -1.55 | -0.28 |
| 8 | 2 | 1280.64 | -28.63 | 1615.88 | 4.66 | -5.69 |
| 9 | 7 | 4642.72 | -91.36 | 5977.76 | 2.89 | -15.85 |
| 10 | 1 | 6347.13 | -74.86 | 8097.93 | -1.53 | -14.97 |

**S-Table 2.** FireDock server generated the top 10 docked models of vaccines to MHC-II.

| **Rank** | [**Solution Number**](http://bioinfo3d.cs.tau.ac.il/FireDock/bin/showRes.pl?id=1kg0MHCII.pdb_Refinedmodel4.pdb_33_37_17_19_3_122&from=1&to=20&sortBy=1) | [**Global Energy**](http://bioinfo3d.cs.tau.ac.il/FireDock/bin/showRes.pl?id=1kg0MHCII.pdb_Refinedmodel4.pdb_33_37_17_19_3_122&from=1&to=20&sortBy=2) | [**Attractive VdW**](http://bioinfo3d.cs.tau.ac.il/FireDock/bin/showRes.pl?id=1kg0MHCII.pdb_Refinedmodel4.pdb_33_37_17_19_3_122&from=1&to=20&sortBy=3) | [**Repulsive VdW**](http://bioinfo3d.cs.tau.ac.il/FireDock/bin/showRes.pl?id=1kg0MHCII.pdb_Refinedmodel4.pdb_33_37_17_19_3_122&from=1&to=20&sortBy=4) | [**ACE**](http://bioinfo3d.cs.tau.ac.il/FireDock/bin/showRes.pl?id=1kg0MHCII.pdb_Refinedmodel4.pdb_33_37_17_19_3_122&from=1&to=20&sortBy=5) | [**HB**](http://bioinfo3d.cs.tau.ac.il/FireDock/bin/showRes.pl?id=1kg0MHCII.pdb_Refinedmodel4.pdb_33_37_17_19_3_122&from=1&to=20&sortBy=6) |
| --- | --- | --- | --- | --- | --- | --- |
| 1 | 8 | -5.21 | -6.29 | 2.42 | 2.93 | 0.00 |
| 2 | 7 | 3.59 | -24.96 | 16.17 | 8.19 | -3.61 |
| 3 | 1 | 5.09 | -9.00 | 10.89 | 0.09 | -1.27 |
| 4 | 3 | 14.22 | -2.61 | 0.00 | 1.90 | 0.00 |
| 5 | 10 | 650.38 | -22.13 | 798.43 | 1.01 | -1.71 |
| 6 | 6 | 1407.51 | -29.11 | 1766.70 | 8.93 | -1.61 |
| 7 | 9 | 2107.50 | -23.82 | 2644.80 | 15.54 | -2.77 |
| 8 | 4 | 2383.41 | -55.39 | 3060.09 | -1.96 | -10.47 |
| 9 | 2 | 6825.84 | -48.65 | 8552.46 | -1.46 | -6.33 |
| 10 | 5 | 10342.20 | -93.52 | 13102.42 | -0.31 | -9.86 |

**S-Table 3.** FireDock server generated the top 10 docked models of vaccines to TLR4.

| **Rank** | [**Solution Number**](http://bioinfo3d.cs.tau.ac.il/FireDock/bin/showRes.pl?id=4g8aTLR4.pdb_Refinemodel.pdb_45_31_8_19_3_122&from=1&to=20&sortBy=1) | [**Global Energy**](http://bioinfo3d.cs.tau.ac.il/FireDock/bin/showRes.pl?id=4g8aTLR4.pdb_Refinemodel.pdb_45_31_8_19_3_122&from=1&to=20&sortBy=2) | [**Attractive VdW**](http://bioinfo3d.cs.tau.ac.il/FireDock/bin/showRes.pl?id=4g8aTLR4.pdb_Refinemodel.pdb_45_31_8_19_3_122&from=1&to=20&sortBy=3) | [**Repulsive VdW**](http://bioinfo3d.cs.tau.ac.il/FireDock/bin/showRes.pl?id=4g8aTLR4.pdb_Refinemodel.pdb_45_31_8_19_3_122&from=1&to=20&sortBy=4) | [**ACE**](http://bioinfo3d.cs.tau.ac.il/FireDock/bin/showRes.pl?id=4g8aTLR4.pdb_Refinemodel.pdb_45_31_8_19_3_122&from=1&to=20&sortBy=5) | [**HB**](http://bioinfo3d.cs.tau.ac.il/FireDock/bin/showRes.pl?id=4g8aTLR4.pdb_Refinemodel.pdb_45_31_8_19_3_122&from=1&to=20&sortBy=6) |
| --- | --- | --- | --- | --- | --- | --- |
| 1 | 5 | -7.65 | -28.83 | 23.61 | 9.17 | -4.34 |
| 2 | 10 | -0.96 | -6.12 | 4.17 | -0.17 | -0.26 |
| 3 | 4 | 5.31 | -23.22 | 5.65 | 14.07 | -5.72 |
| 4 | 2 | 5.65 | -1.00 | 0.00 | 0.18 | -0.62 |
| 5 | 3 | 6.95 | -7.89 | 2.99 | 3.70 | -1.18 |
| 6 | 8 | 13.28 | -7.24 | 3.70 | 4.04 | 0.00 |
| 7 | 7 | 25.44 | -29.93 | 20.81 | 16.33 | -2.89 |
| 8 | 6 | 26.70 | -12.97 | 14.29 | 11.35 | -3.81 |
| 9 | 9 | 520.23 | -24.10 | 660.66 | 14.20 | -3.40 |
| 10 | 1 | 7838.98 | -107.48 | 10005.12 | 6.33 | -10.08 |
